# Hyperbaric oxygen therapy improves post-concussion symptoms in adults with childhood traumatic brain injury: a retrospective cohort study

**DOI:** 10.3389/fneur.2025.1641033

**Published:** 2025-09-03

**Authors:** Adi Shabi Shlifer, Gil Suzin, Ran Shorer, Erez Lang, Shachar Finci, Karin Elman-Shina, Keren Doenyas-Barak, Shai Efrati

**Affiliations:** ^1^Sagol Center for Hyperbaric Medicine and Research, Shamir, Medical Center, Be’er Ya’akov, Israel; ^2^Gray School of Medicine, Faculty of Medical and Health Sciences, Tel Aviv University, Tel Aviv, Israel; ^3^Sagol School of Neuroscience, Tel Aviv University, Tel Aviv, Israel

**Keywords:** hyperbaric oxygen therapy, traumatic brain injury, post-concussion syndrome, pediatric brain injury, cognitive rehabilitation, neuroplasticity

## Abstract

Post-concussion syndrome (PCS) following childhood traumatic brain injury (TBI) can result in persistent cognitive impairments that extend into adulthood, yet it remains significantly underdiagnosed and undertreated. This study evaluated the effects of hyperbaric oxygen therapy (HBOT) on chronic neurocognitive symptoms in adults with PCS stemming from pediatric TBI. We conducted a retrospective analysis of patients treated with HBOT at the “Sagol Center for Hyperbaric Medicine and Research” between 2017 and 2024. Inclusion criteria included TBI before age 17, HBOT initiation after age 20, and the availability of computerized cognitive assessments before and after treatment. All participants received at least 40 sessions of HBOT, consisting of 90 min of 100% oxygen at 2 ATA with air breaks. Twenty-six adults (mean age 31.7 ± 8.6 years) who sustained TBI in childhood (mean age at injury 7.7 ± 5.8 years) met inclusion criteria. Following HBOT, statistically significant improvements were observed in all cognitive domains except for motor skills (global cognition, memory, executive function, attention and information processing speed; all adjusted *p* < 0.05; effect sizes r = 0.62–0.78, Wilcoxon signed-rank test). These improvements were independent of time since injury (mean 23.6 ± 9.3 years) and initial TBI severity. Notably, individuals with a history of mild TBI exhibited similar impairments and treatment response to those with more severe injuries. These findings suggest that HBOT may induce meaningful neurocognitive improvement even decades after pediatric TBI, supporting its potential role in long-term rehabilitation strategies for this underserved population.

## Introduction

Traumatic brain injury (TBI), a leading cause of emergency department visits in children, has an estimated annual incidence of 150 to 400 cases per 100,000 in the pediatric population (1). Although most pediatric TBIs are classified as mild (mTBI), a substantial proportion of affected children—estimated between 10 and 30%—develop persistent post-concussion syndrome (PCS), characterized by long-term cognitive, emotional, somatic and behavioral symptoms. These symptoms may persist for years and interfere with academic and social functioning (2–6).

Despite increasing awareness, PCS in children continues to be overlooked by clinicians and educators. Many children experiencing long-term post-injury symptoms do not receive a formal diagnosis or appropriate interventions, allowing their difficulties to persist untreated into adulthood (7).

Hyperbaric oxygen therapy (HBOT) has recently gained attention as a potential treatment for chronic PCS (8–12). By increasing oxygen delivery under elevated pressure, dedicated protocols of HBOT may induce neuroplasticity and recovery of dysfunctional brain regions even at the late chronic phase (13, 14). Clinical studies have demonstrated improvements in cognitive and behavioral outcomes following HBOT in both pediatric and adult populations with PCS (8–11, 14). However, the therapeutic impact of HBOT on adults suffering from chronic PCS resulting from childhood TBI has not yet been investigated.

The present study aims to address this gap by evaluating the effects of HBOT in adults with longstanding PCS following pediatric TBI. Using objective computerized neurocognitive assessments, we investigated whether a standardized HBOT protocol could induce measurable cognitive improvements in this unique and underserved population.

## Materials and methods

### Participants

**Figure 1:**
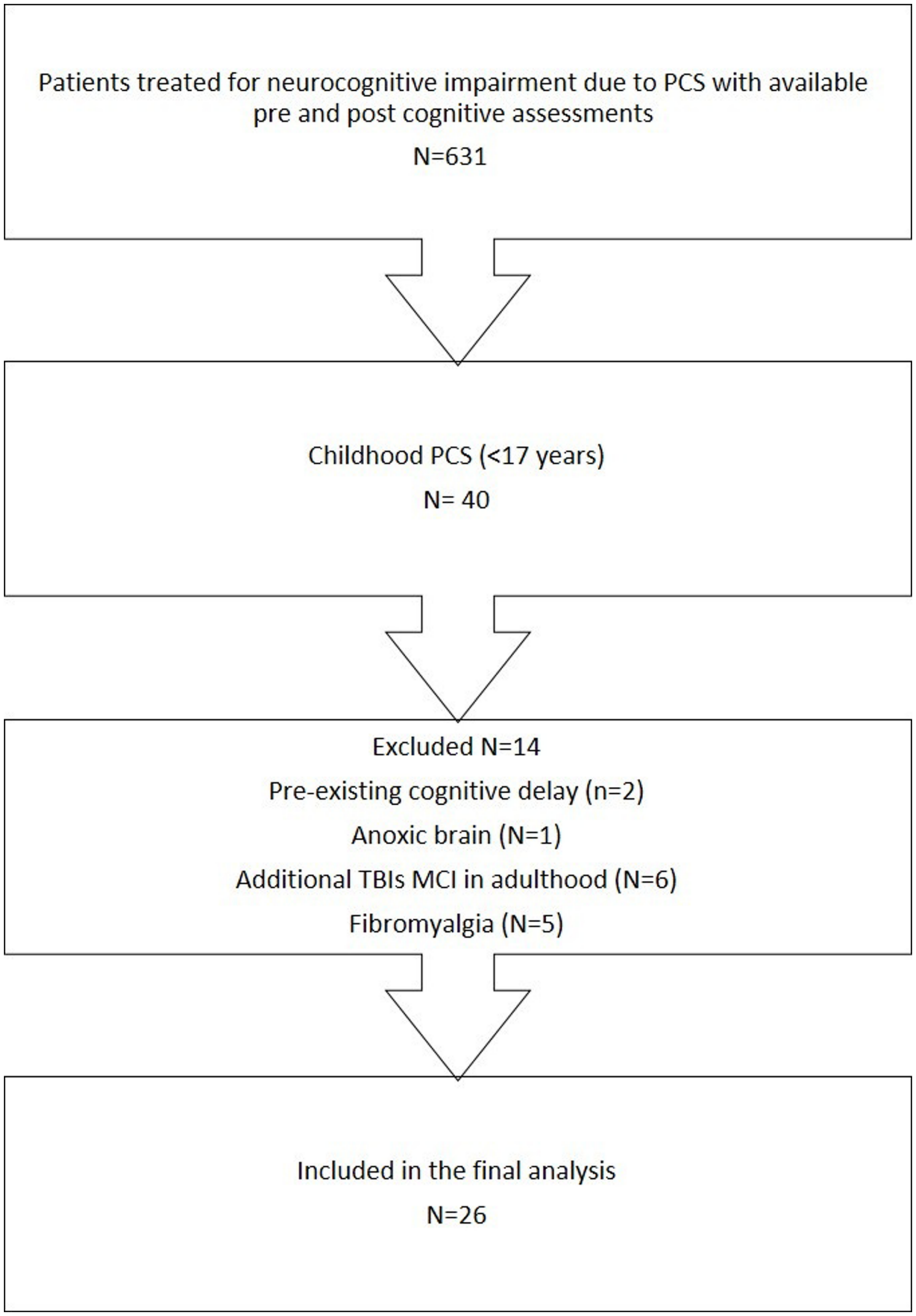
Patient inclusion flow chart depicting the screening and exclusion process leading to the final study sample (*N* = 26).

This retrospective study included patients with chronic neurocognitive symptoms due to post-concussion syndrome (PCS) following traumatic brain injury (TBI), treated at the Sagol Center for Hyperbaric Medicine and Research, Shamir Medical Center, Israel, between 2017 and 2024. Eligible participants had sustained TBI before the age of 17 and received hyperbaric oxygen therapy (HBOT) as adults (age > 20 years). Inclusion criteria required the availability of pre-and post-treatment computerized cognitive assessments. Exclusion criteria included history of anoxic brain injury, pre-existing cognitive impairment, or other neurological conditions that could confound the diagnosis. All patients meeting the inclusion criteria were consecutively included in the study analysis (see Figure 1 for patient flow).

PCS was diagnosed according to ICD-11 criteria, requiring (1) a documented mTBI or concussion; (2) persistence of symptoms beyond the typical recovery window (>14 days in adults or >28 days in children); and (3) objective evidence of functional impairment (15). Clinical evaluation considered four domains: cognitive, somatic, emotional/behavioral, and sleep–wake disturbances. The classification of TBI severity (mild vs. moderate–severe) was based on clinical records, including available data on acute hospitalization, neuroimaging findings, and neurological assessments documented at the time of injury, such as Glasgow Coma Scale (GCS) scores when available. Severity classification followed standard criteria, with mild TBI defined by a GCS score of 13–15, brief or no loss of consciousness, and normal neuroimaging when available. Moderate-to-severe TBI included cases with GCS scores below 13, evidence of intracranial pathology on imaging, prolonged loss of consciousness, or other clinical indicators consistent with greater injury severity.

### Ethical approval

The study was approved by the Institutional Review Board (IRB) of Shamir Medical Center. All procedures conformed to the ethical standards outlined in the Declaration of Helsinki.

### Hyperbaric oxygen therapy protocol

Patients underwent at least 40 consecutive HBOT sessions, delivered five times per week. Each session lasted 90 min and involved breathing 100% oxygen at 2 atmospheres absolute (ATA), with 5-min air breaks every 20 min.

### Cognitive assessment

Cognitive performance was assessed using the NeuroTrax computerized battery (NeuroTrax Corp.), which evaluates memory (verbal/non-verbal, immediate/delayed), executive function, attention, information processing speed and motor skills. The cognitive indices were based on scores of six cognitive tests: verbal memory, non-verbal memory, go-no-go test, Stroop test, staged information processing test and catch game. A global cognitive score was computed as the average of all domain scores. Scores were normalized to an IQ-like scale (mean = 100, SD = 15), adjusted for age and education, based on a normative database from over 10 research sites (16, 17). Additional information is also available on the NeuroTrax website.^1^

To minimize learning effects, equivalent but non-identical test versions were used for pre-and post-HBOT evaluations. These have demonstrated high test–retest reliability and minimal practice effects (16). Prior validation studies, including a randomized controlled trial in a similar population, showed score stability in untreated controls (18).

All cognitive assessments were administered in a quiet clinical setting using standardized computer-based testing procedures. Pre-treatment assessments were performed within 2 month prior to initiating HBOT, and post-treatment assessments were conducted within 1 month after completing the last HBOT session.

### Statistical analysis

Shapiro–Wilk tests revealed that cognitive outcome data were not normally distributed. Therefore, Wilcoxon signed-rank tests were used to compare pre-and post-treatment scores. Subgroup analyses by TBI severity (mild vs. moderate-to-severe) were conducted using Mann–Whitney U tests. Associations between time since injury and cognitive improvement were examined using Spearman’s rank correlation. Effect sizes were calculated using the rank-based effect size r (Z/√N). All tests were two-tailed, with significance set at *α* = 0.05, and *p*-values for the six cognitive outcomes were adjusted for multiple comparisons using the Bonferroni correction.

## Results

### Patient selection and characteristics

Between 2017 and 2024, 631 patients with neurocognitive symptoms due to post-concussion syndrome (PCS) were treated at the Sagol Center for Hyperbaric Medicine and Research. Of these, 40 had sustained traumatic brain injury (TBI) before the age of 17. Fourteen patients were excluded: two due to pre-existing cognitive delays, one due to an anoxic brain injury preceding the TBI, six due to additional TBIs or development of mild cognitive impairment (MCI) in adulthood, and five due to co-morbid fibromyalgia. A total of 26 patients met all inclusion criteria and were included in the final analysis. No adverse events or safety concerns were observed during or following the HBOT sessions. The patient inclusion process is summarized in Figure 1.

Baseline characteristics are presented in Table 1. Data are presented as mean ± standard deviation (SD) unless otherwise specified. The average age at the time of treatment was 31.7 ± 8.6 years; 77% were male. The mean age at injury was 7.7 ± 5.8 years, and the average time elapsed from injury to treatment was 23.6 ± 9.3 years (range: 3–35 years). Thirteen participants (50%) sustained mild TBI and 13 (50%) moderate TBI. All patients reported persistent cognitive symptoms attributed to their childhood TBI. On average, participants completed 60.0 ± 1.3 HBOT sessions (range: 40–70 sessions).

**Table 1:** Baseline demographic and clinical characteristics of participants by TBI severity subgroup (mild vs. moderate).

Characteristics	Total	Mild TBI	Moderate TBI
Patients (n)	26 (100%)	13 (50%)	13 (50%)
Age (years)	31.7 ± 8.6	33 ± 8.7	30.4 ± 8.7
Men	20 (77%)	9 (45%)	11 (55%)
Women	6 (23%)	4 (67%)	2 (33%)
Education (years)	13.2 ± 1.7	13.6 ± 1.4	12.8 ± 2
Traumatic event			
Motor vehicle accident	9 (36%)	1 (11%)	8 (88%)
Fall	13 (50%)	10 (77%)	3 (23%)
Blow	3 (12%)	2 (67%)	1 (33%)
Age at the event (years)	7.7 ± 5.8	6 ± 5.6	9.3 ± 5.8
Time from trauma (years)	23.6 ± 9.3	26 ± 8	21.2 ± 10
Symptoms			
Cognitive	26 (100%)	13 (100%)	13 (100%)
Motor	11 (42%)	1 (9%)	10 (91%)
Emotional	12 (46%)	6 (50%)	6 (50%)
Sensory	11 (42%)	6 (55%)	5 (45%)
HBOT sessions	60.04 + −1.3	60	59.6

Values are presented as mean ± SD or *n* (%), unless otherwise specified.

### Neurocognitive outcomes

**Figure 2:**
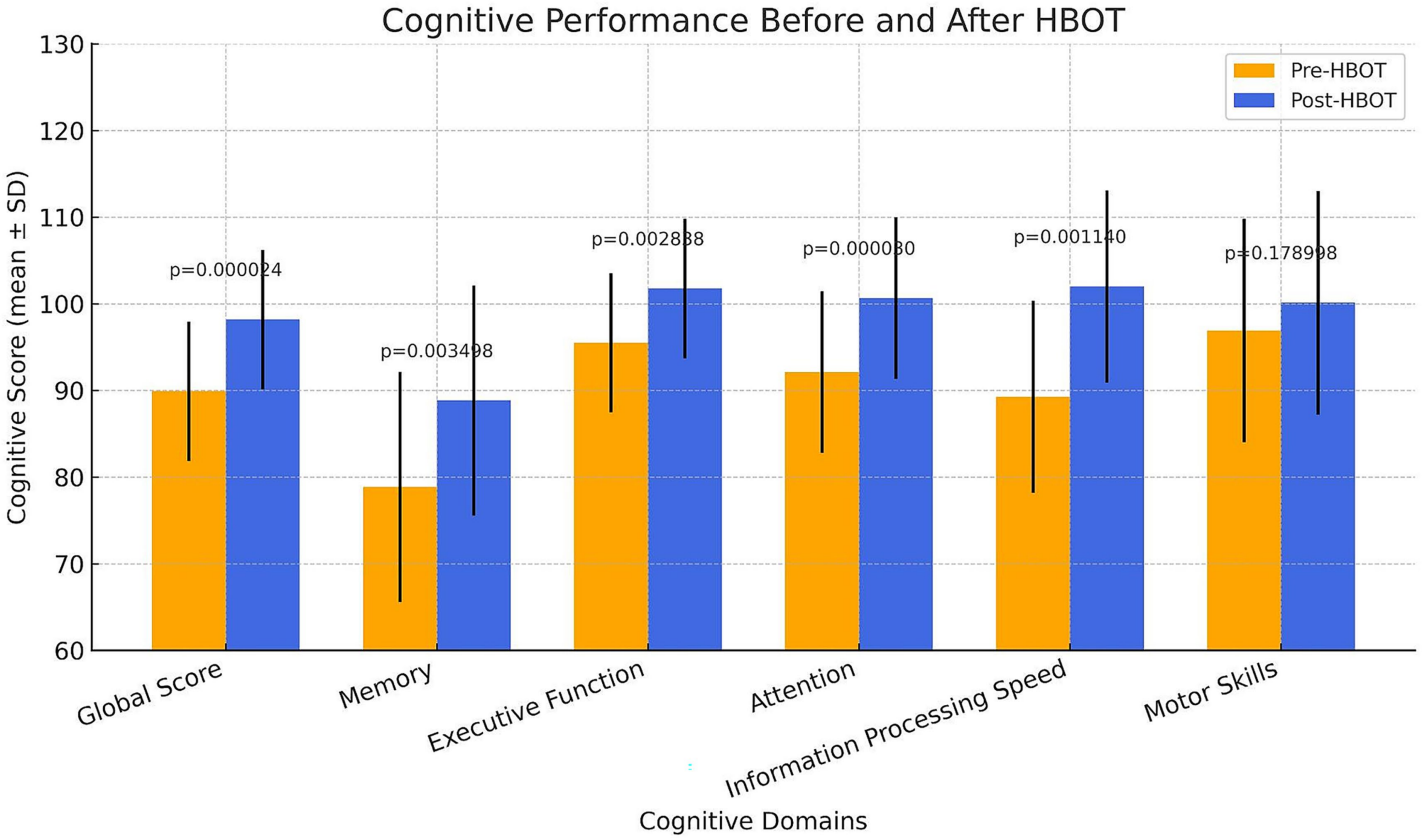
Cognitive scores before and after HBOT across six domains. Bars represent means ± standard deviations for pre- and post-treatment scores. Statistically significant improvements were observed in all domains except for Motor Skills (adjusted *p* < 0.05, Wilcoxon signed-rank test, with p-values adjusted for multiple comparison using the Bonferroni correction).

The neurocognitive outcomes are summarized in Table 2 and visualized in Figure 2. After Bonferroni correction for multiple comparisons, significant improvements were observed in all cognitive domains except Motor Skills (*N* = 26).

- *Global Cognitive Function* improved by 8.06 ± 6.97 points (*p* = 0.000024; *r* = 0.779).
- *Memory Function* improved by 9.82 ± 13.63 points (*p* = 0.003498; r = 0.620).
- *Executive Function* improved by 5.94 ± 8.88 points (*p* = 0.002838; r = 0.628).
- *Attention* improved by 8.50 ± 9.09 points (*p* = 0.00003; r = 0.775).
- *Information Processing Speed* improved by 9.84 ± 12.31 points (*p* = 0.00114; r = 0.685).
- *Motor Skills* improved by 3.10 ± 7.47 points (*p* = 0.178998; r = 0.409).

**Table 2:** Cognitive outcomes before and after HBOT.

Cognitive domain	Pre HBOT (Mean ± SD)	Post HBOT (Mean ± SD)	Δ (Mean ± SD)	*p*-value (original)	*p*-value (Bonferroni)	Effect size (r)
Global Score	87.42 ± 21.90	95.48 ± 22.61	8.06 ± 8.07	0.000004	0.000024	0.779
Memory	77.17 ± 26.97	86.99 ± 25.78	9.82 ± 13.26	0.000583	0.003498	0.620
Executive Function	92.82 ± 21.77	98.76 ± 23.59	5.94 ± 7.92	0.000473	0.002838	0.628
Attention	89.26 ± 20.82	97.76 ± 21.62	8.50 ± 9.33	0.000005	0.00003	0.775
Information Processing Speed	88.60 ± 23.53	98.44 ± 25.73	9.84 ± 11.04	0.000190	0.00114	0.685
Motor Skills	94.20 ± 24.44	97.30 ± 25.05	3.10 ± 12.66	0.029833	0.178998	0.409

Values are presented as means with standard deviations (Mean ± SD). Δ represents the mean change in standardized scores with its standard deviation. *P*-values were derived from Wilcoxon signed-rank tests and adjusted for multiple comparisons using the Bonferroni correction. Effect sizes (r) were calculated as Z divided by the square root of the sample size.

### Time since trauma and treatment response

Spearman correlation analyses revealed weak-to-moderate negative correlations between time since injury and cognitive improvement across cognitive domains (r = −0.02 to −0.33; all *p* > 0.1), suggesting that the time elapsed since trauma did not significantly influence treatment efficacy.

### Impact of TBI severity

Both mild and moderate TBI subgroups demonstrated significant cognitive improvements following HBOT. Although numerically different gains were observed across domains, Mann–Whitney U tests revealed no statistically significant differences in cognitive improvement between the groups (all *p* > 0.12). These results suggest that the observed cognitive benefits were largely independent of initial TBI severity.

## Discussion

This study is the first to systematically evaluate the effects of HBOT in adults with chronic longstanding neurocognitive impairments stemming from post-concussion syndrome (PCS) following childhood traumatic brain injury (TBI). Statistically significant improvements were observed across all major cognitive domains, including global cognition, memory, executive function, attention, and information processing speed. Effect sizes ranged from medium to large (*r* = 0.62–0.78), reinforcing the clinical relevance of the observed outcomes.

In this study, we aimed to specifically assess the neurocognitive effect of HBOT on adults with persistent PCS stemming from childhood TBI, without confounding neurological or developmental conditions. Therefore, we applied strict inclusion criteria, selecting only individuals with a documented TBI before age 17, no additional acquired or congenital brain injuries, and sufficient physical and cognitive stability to complete a full course of at least 40 HBOT sessions. This targeted approach allowed us to isolate the effects of HBOT on this unique population and provides initial insights into its potential role in long-term neurorehabilitation.

Importantly, treatment effectiveness was not significantly associated with time elapsed since injury. With time from trauma ranging widely—from as little as 3 years to as long as 37 years post-injury (mean: 23 years)—these findings underscore the potential of HBOT to promote meaningful neuroplasticity and cognitive recovery even decades after the initial trauma. While numerically different gains were observed between severity groups, there was no statistically significant differences in cognitive improvement between patients with mild and those with moderate TBI (all *p* > 0.12). The findings in this study on the beneficial effect of HBOT on prolonged PCS are in line with other studies where treatment was initiated even years after the acute insult (8, 9, 14, 19).

The observed improvements may be attributed to the underlying biological mechanisms of HBOT. By dedicated protocols of pressure and oxygen fluctuations, HBOT enhances tissue oxygenation and triggers regenerative pathways through oxygen and pressure sensitive gene expression, a phenomenon known as the Hyperoxic-Hypoxic Paradox (HHP) (13). These fluctuations activate cascades of biological effects that modulate inflammatory pathways, restore mitochondrial function, proliferation and migration, promote angiogenesis, and stimulate the proliferation and migration of neural stem cells. Given the role of chronic neuroinflammation and vascular dysfunction in prolonged PCS, these mechanisms offer a plausible basis for recovery observed even decades after TBI (14, 20–22).

Notably, while a growing body of evidence supports the benefits of HBOT for PCS, the literature has also revealed heterogeneity in outcomes. As emphasized by Harch et al., Borlongan et.al., and Hadanny et al. (23–25), such variability may be attributed to methodological differences across studies, including variations in treatment protocols, control conditions, study endpoints and patient populations. These inconsistencies highlight the critical importance of employing dedicated and standardized HBOT protocols that carefully calibrate oxygen pressure, exposure duration, and total number of sessions. The current study implemented such a standardized protocol, which may have contributed to the robust cognitive improvements observed in this uniquely underrepresented population of adults with childhood-onset TBI.

An expanding body of clinical research, including well-controlled trials, continues to demonstrate the potential of HBOT to improve neurocognitive functions in patients with chronic PCS. In a double-blind, sham-controlled trial, children treated with HBOT exhibited significant improvements in memory, executive function, and behavior, accompanied by structural brain changes detected by neuroimaging (10).

Similarly, studies in adults have demonstrated durable enhancements in cognitive function and cerebral perfusion following 40 to 80 sessions of HBOT (8, 26). Boussi-Gross et al. (9) demonstrated significant cognitive and quality-of-life improvements in adults with PCS treated with 60 HBOT sessions, supported by perfusion imaging changes. Tal et al. (27) observed improved executive function, attention, and cerebral blood flow in veterans with chronic mild TBI following HBOT. In a recent double-blind randomized trial, adults with persistent post-traumatic symptoms showed significantly greater improvements in neurobehavioral outcomes after 40 sessions of HBOT compared to sham treatment, with additional gains observed following a second treatment series at 12 months (11). Other studies have reported neuroplastic changes and normalization of functional brain connectivity after treatment, further reinforcing the therapeutic potential of HBOT in chronic brain injury (28–30).

However, to our knowledge, the present study is the first to systematically investigate HBOT outcomes in adults with PCS resulting from TBI sustained during childhood. This population is rarely addressed in HBOT literature despite representing a unique clinical profile shaped by early developmental-stage injury. The findings extend the current evidence base by demonstrating that meaningful cognitive recovery can occur decades after childhood TBI, independent of initial injury severity, when appropriate neuroregenerative treatment is applied.

### Clinical implications

A particularly notable finding is the significant benefit observed in patients with a history of mild TBI. Despite often being excluded from structured rehabilitation programs, individuals with mild injuries may suffer from persistent deficits that affect their academic, occupational, and emotional development (31). In this study, patients with mild TBI demonstrated improvements in multiple cognitive domains, underscoring the need to reassess assumptions regarding the benign nature of mild pediatric TBI. Furthermore, nearly half of the patients reported additional emotional (46%) and sensory (42%) symptoms, indicating the broader impact of childhood TBI on adult function.

The treatment protocol used an average of 60 HBOT sessions—was tailored to support time-dependent regenerative processes. Vascular remodeling, angiogenesis, and neurogenesis require sustained stimulation, and studies suggest that at least 40–60 sessions are needed to initiate and consolidate neurovascular integration (24). Our regimen aligns with this understanding and reflects emerging evidence on the minimal number of sessions for HBOT in chronic neurological conditions (11, 20, 24, 32, 33).

### Study limitations

This study is limited by its retrospective design, lack of a control group, and single-center setting. While the use of standardized, age-and education-adjusted computerized assessments strengthens internal validity, generalizability may be constrained. However, previous validation studies using the NeuroTrax battery, particularly with alternate test versions have demonstrated high reliability and minimal practice effects, supporting the credibility of the results (34).

The study’s relatively small sample size and highly selective inclusion criteria limit the generalizability of the findings. Participants were individuals with no other acquired or congenital neurological conditions, and all were able to complete at least 40 HBOT sessions. As such, the results should be interpreted cautiously when considering broader or more heterogeneous PCS populations. Nevertheless, other studies have demonstrated significant HBOT-related cognitive improvements in more diverse TBI cohorts, supporting the relevance of our findings within the broader context of neurorehabilitation (4, 5, 8–11, 13, 26).

While the current study focused on objective cognitive outcomes, clinical impressions documented during follow-up suggest that participants often experienced broader symptom improvements, including enhanced mood, sleep quality, and daily functioning. Although these domains were not formally assessed in this analysis, future studies should incorporate validated patient-reported outcome measures to comprehensively evaluate the clinical impact of HBOT on post-concussion syndrome.

Additional limitation is related to the lack of long term follow up. Pre-treatment evaluations were conducted within 2 months prior to initiating HBOT, and post-treatment assessments were performed within 1 month following the final session. As the study focused solely on the immediate post-treatment period, the findings do not allow us to confirm the long-term persistence of the observed cognitive improvements. Future studies should incorporate extended follow-up assessments to evaluate the long-term durability of these effects.

## Conclusion and future directions

This study provides initial evidence that HBOT can elicit significant cognitive improvements in adults with chronic PCS from childhood TBI. Given that treatment effects were not dependent on time since injury or injury severity, HBOT may represent a valuable therapeutic option even for those with longstanding symptoms. Future prospective, controlled trials are warranted to confirm these findings, elucidate optimal dosing protocols, and explore adjunctive interventions and assess the long-term durability of treatment effects through extended follow-up. These results also highlight the need for early recognition, long-term follow-up, and access to rehabilitation for pediatric TBI survivors, especially those with mild injuries who are often misclassified and undertreated.

## Data Availability

The data analyzed in this study is subject to the following licenses/restrictions: the dataset analyzed in this study contains sensitive patient information and is not publicly available due to privacy and ethical restrictions. Access to the data may be granted upon reasonable request and approval by the institutional review board (IRB) and data protection authorities. Requests to access these datasets should be directed to AS, adishabi@gmail.com.

## Footnotes

^1^ http://www.neurotrax.com/
